# Removing infrarenal inferior vena cava filters (IVCFs) with thrombus under protection of suprarenal IVCFs: A retrospective study in a single-center institution

**DOI:** 10.1097/MD.0000000000035574

**Published:** 2023-10-20

**Authors:** Pengkai Cao, Xintong Luo, Yunsong Li, Xiangdong Liu, Liang Li, Yaodong Dou, Yanrong Zhang

**Affiliations:** a Vascular Surgery Department of Third Hospital of Hebei Medical University, Shijiazhuang, China; b Neurology Department of Hebei General Hospital, Shijiazhuang, China.

**Keywords:** anticoagulation, inferior vena cava filters, infrarenal, retrieval, suprarenal, thrombus

## Abstract

To determine feasibility of removing inferior vena cava filters (IVCFs) with massive thrombus (>1*1cm) under protection of suprarenal IVCFs, and evaluate the filter thrombus detachment due to removal. The patients who had massive infrarenal IVCFs thrombus and received retrieval under protection of suprarenal IVCFs were retrospectively reviewed from July 2018 to December 2021. Medical data of them including demographics, filter types, dwell time, management, thrombus detachment was collected, and analyzed. There were 33 patients having massive infrarenal IVCFs thrombus and receiving retrieval under protection of suprarenal IVCFs including 23 males and 10 females with a mean age of 55.30 ± 11.97 (range, 30–85 years). All Infrarenal IVCFs were removed successfully and 29 cases (87.88%) were confirmed detachment of thrombus by cavography including 7 small-size thrombus (<1*1cm) and 22 large-size thrombus (>1*1cm). Twenty-two suprarenal IVCFs trapped large-size thrombus were treated with additional anticoagulation and 21 of them had successful retrievals with additional anticoagulation period of 66.18 ± 43.38 days (range, 9–154 days). The large-size IVCFs thrombus may be break off during retrieval, and IVCFs with large-size thrombus could be removed safely with suprarenal IVCFs protection. The thrombus trapped in filters could be reduced with an additional period of anticoagulation.

## 1. Introduction

Retrievable Inferior vena cava filters (IVCFs) have become one of the main treatments for venous thromboembolism patients who had contraindications to anticoagulation in last decade.^[1–4]^ Although they were designed to be implanted for reducing the incidence of pulmonary embolism (PE) and then be retrieved as the protection no longer required,^[5,6]^ numbers of IVCFs were not removed and the retrieval rate kept in consistently low levels.^[7–9]^ Complications including filter fractures, penetration, migration, occlusions of inferior vena cava (IVC) has been emerged with the dramatic rise in permanent filter placement.^[7,9–12]^ Despite lots of efforts focused on improving retrieval rate in recent years,^[13–15]^ some issues remain unresolved.

Filter thrombus, usually discovered at the time of retrieval with an incidence ranged from 6 to 81%,^[15–21]^ may not be associated with a worse prognosis,^[20]^ but it still was a common cause of IVCF retrieval failure. IVCFs with small-size thrombus (<1*1cm) could be removed using regular techniques,^[15,18,22]^ minimal study is available on filters with massive thrombus (>1*1cm) yet. Procedures of retrieval may be directly withdrawn as thrombus within filter were confirmed by venography in the past.^[15,18,22]^ Catheter directed thrombolysis (CDT) and pharmaco-mechanical thrombectomy (PMT) could be performed to reduce thrombus burden within the filter,^[23]^ but the chances that the volume of residual thrombus is still > 1*1cm after CDT/PMT or contraindications of CDT/PMT exist mayn’t be avoided.

The present study attempted to determine feasibility of removing IVCFs with massive thrombus under protection of suprarenal IVCFs, and evaluate the filter thrombus detachment due to removal.

## 2. Patients and Methods

After approval obtained by the Third Hospital of Hebei Medial University Institutional Committee of Ethics in Research (W2023-071-1), patients receiving IVCFs placement and retrieval at Third Hospital of Hebei Medial University from July 2018 to December 2021 were retrospectively reviewed. The patients who had massive intra-filter thrombus after CDT/PMT or had contraindications of CDT/PMT were selected. Medical data of them including demographics, baseline medical history, types of infrarenal and suprarenal IVCFs, dwell time of infrarenal, and suprarenal IVCFs, management for IVCF thrombus, whether there was thrombus detachment due to infrarenal IVCFs removal, duration of anticoagulation before suprarenal IVCFs retrieval, as well as technical success rate of suprarenal IVCFs removal.

### 2.1. Managements for IVCFs thrombus

When IVCFs thrombus were detected during retrieval, thrombus was classified based on size (the longest longitudinal length and the widest perpendicular width were measured on the anteroposterior cavography at filter removal) as small (<1*1cm), large (>1*1cm). IVCFs with small-size thrombus were retrieval directly without additional treatment. If there was no contraindication, CDT/PMT would be done for large thrombus within IVCFs. As the thrombus size decreased to < 1*1cm, retrieval was performed. But if the volume of residual colt was still > 1*1cm after CDT/PMT or contraindications of CDT/PMT existed, suprarenal IVCFs would be considered.

### 2.2. Suprarenal IVCFs placement

Venous access was obtained via the right internal jugular vein, and then cavography was performed through a 5F pigtail catheter to access the IVC anatomy and IVCFs thrombus. After conforming the location of left renal vein, 5F pigtail catheter was exchanged over a 0.035 guidewire for the filter delivery sheath, and the filter was deployed with the lower edge of filter above the left renal vein. Repeat cavography was performed after filter implantation.

### 2.3. Infrarenal IVCFs with massive thrombus removal

Infrarenal IVCFs retrieval was attempted in patients who had received suprarenal IVCFs implantation. If possible, a 12F delivery sheath should be considered to be used in the procedures. After the snare caught the hook of IVCFs, the filter was slowly withdrawn into the sheath and then taken out. Meanwhile a 20 mL syringe was applied to extract from the side port of the sheath in order to make sure there was no thrombus in the sheath.

### 2.4. Suprarenal IVCFs retrieval

Repeat cavography was performed to exclude IVC injury and thrombus detachments trapped by suprarenal IVCFs were analyzed. If no thrombus trapped within suprarenal IVCFs or thrombus < 1*1 cm, suprarenal IVCFs were removed immediately. If thrombus trapped within suprarenal IVCFs > 1*1 cm, oral anticoagulation (Rivaroxaban 20 mg 1/day) was prescribed and regular follow-up checks were performed by ultrasonography or venography. Suprarenal IVCFs retrieval procedure was performed by routine as the thrombus decreased to < 1*1 cm or disappeared.

### 2.5. Statistical analysis

Statistical analysis was performed by SPSS 22.0 software (IBM Corp, Armonk, NY). The normally distributed quantitative variables were presented as means + standard deviation, and when variables were not normally distributed, the interquartile range was used. Nominal data were reported as the number of subjects or incidence rates, and Wilcoxon–Mann–Whitney test was used to test their difference. Statistical significance was defined as *P* < .05.

## 3. Results

From July 2018 to December 2021, there were 33 cases of large-size IVCFs thrombus who suffered the volume of residual colt was still > 1*1cm after CDT/PMT or had contraindications of CDT/PMT. Demographics, baseline medical history and IVCFs placement indications of 23 male patients and 10 female patients with a mean age of 55.30 ± 11.97 (range, 30–85 years) were summarized in Table 1.

**Table 1:** Clinical characteristics of the patients and indications for IVCFs placement.

Gender	
Male	23
Female	10
Age	55.30 ± 11.97 (range, 30–85 yr)
Body mass index	26.71 ± 4.22
Hemoglobin (g/L)	
At ICVFs implantation	120.38 ± 20.12
At ICVFs removal	122.66 ± 15.85
Platelet (10^9/L)	
At ICVFs implantation	179.50 (IQR 146.10–251.15)
At ICVFs removal	237.80 (IQR 164.00–204.30)
Cardiovascular risk factor	
Arrhythmias	2
Hypertension	11
Coronary heart disease	3
Nephrotic syndrome	1
Diabetes	3
Primary affections	
Severe trauma	5
Traumatic intracranial haemorrhge	3
Lower limb fracture	16
Spine fracture	1
Lumbar stenosis	4
Nephrotic syndrome	1
Hyperhomocysteinemia	1
May-Thurner syndrome	1
Others	1
Indications of IVCFs	
DVT with contraindication to anticoagulation	22
Traumatic intracranial hemorrhage	3
Severe trauma	5
Emergency surgery or planned < 72 h	11
High risk of bleeding	3
Adjunct to thrombolysis	3
Large, free-floating proximal DVT	7
Recurrent DVT despite adequate anticoagulation	1

IVCFs = inferior vena cava filters.

List in Table 2, 20 of 33 patients received CDT/PMT with a failure of thrombus regression including 17 cases of residual colt still > 1*1cm, 1 cases of low fibrinogen level, and 2 cases of bleeding events. Two patients refused CDT/PMT for personal reasons and the other 11 of 33 patients had contraindications to CDT/PMT like major surgery, recent intracranial trauma, or major trauma.

**Table 2:** Reasons for infrarenal IVCFs retrieval with protection of suprarenal IVCFs.

	NO.
Failure of thrombus regression	
Residual colt > 1*1 cm	17
Low fibrinogen level	1
Bleeding event	2
Contraindication to CDT/PMT	
Major surgery	2
Recent intracranial haemorrhge	3
Recent major trauma	5
Old age (>75 yr)	1
Refuse to CDT/PMT	2

CDT = catheter directed thrombolysis, IVCFs = inferior vena cava filters, PMT = pharmaco-mechanical thrombectomy.

All Infrarenal IVCFs of 33 patients were removed successfully and no clinically symptomatic PE (no patients had signs and symptoms including sudden dyspnea, chest pain, syncope or dizziness) or other complications occurred. The detail of filter types, dwelling time were showed in Table 3. During the study period, a total of 29 cases were confirmed detachment of thrombus after infrarenal IVCFs retrieval including 7 small-size thrombus and 22 large-size thrombus with an incidence rate 87.88%, and no occlusion of suprarenal IVC occured. Eleven suprarenal IVCFs trapped small-size thrombus or no thrombus were removed immediately. Twenty-two suprarenal IVCFs trapped large-size thrombus were removed after additional anticoagulation. There was 1 suprarenal IVCF (Cordis Optease, Cordis Medical Device Co. Florida) found out containing large-size thrombus and then receiving another CDT before retrieval. Just because of this case, later in present study operators preferred conical-shaped filters at suprarenal positon which may be seen in Table 2. The majority of infrarenal IVCFs were double-basket-design filters including 17 cases of Optease, and 13 cases of Aegisy (LifeTech Scientific Corporation, Shenzhen, China). While most of suprarenal IVCFs were conical-shaped filters containing 5 cases of Celect (Cook medical, Bloomington), 16 cases of Danali (Bard Peripheral Vascular Inc. Tempe) and 1 case of Option (Argon Medical Devices Inc. Athens).

**Table 3:** Types, dwelling time and thrombus size of IVCFs.

	Infrarenal IVCFs	Suprarenal IVCFs
Filters type		
Denali	2	16
Celcet	1	5
Option	0	1
Optease	17	3
Aegisy	13	8
Dwelling time	18.30 ± 16.81	49.21 ± 49.56
Removing immediately	0	11
<14 d	8	3
>14 d but < 30 d	23	2
>30 days but < 90 d	1	15
>90 d	1	2
Thrombus size		
<1*1 cm (small-size)	0	7
>1*1 cm (large-size)	21	22
Occlusion completely	12	0

IVCFs = inferior vena cava filters.

There were 22 suprarenal IVCFs trapped large-size thrombus having additional anticoagulation for a mean period of 66.18 ± 43.38 days (range, 9–154 days) before retrieval. Those suprarenal IVCFs of 22 patients were removed successfully except 1 suprarenal IVCF. The unretrieved suprarenal IVCF (Cook Celect) was tiled and embedded, we tried several measures and it could be catch via “loop” technique. But when we tried to remove it, the patient complained unaccepted pain and gave up retrieval. The total successful rate of suprarenal IVCFs retrieval was 96.97% (32/33), and no symptomatic PE or other complications occurred either.

## 4. Discussion

The incidence of filter thrombus varied by many factors including indwelling time, study population, filter types etc.^[18,19,21,24]^ Moreover, higher rates seemed to reported in double-basket-design filters^[18,25]^ like Optease, Aegisy, whose recommended optimal removal time were within 12 to 14 days. Most IVCFs thrombus in present study were found in double-basket-design filters which may be consistent with those research. For this kind filters, increased indwelling time would be accompanied by complex removal procedure or failure of IVCFs retrieval,^[26]^ so most managements of massive filter thrombus were left IVCFs as permanent devices or retrieved after clot burden reduction.^[27]^

Based on a former vitro model, the mass of thrombus retrieved with filters was only a fraction of the initial clot burden.^[28]^ The present study detected that removing IVCFs with large-size thrombus may result in thrombus detachment with a high incidence rate. Early discovery, diagnosis and treatment may play a vital role in increasing IVCFs retrieval rate. Except complete IVC occlusion, IVCFs thrombus were mostly asymptomatic which only were discovered at retrieval, and it is generally difficult to identify the source of IVCFs thrombus which is trapped emboli or filter induced thrombosis.^[29]^ Although IVCFs thrombus may not be associated with a worse prognosis,^[20]^ higher mortality and significant morbidities could be caused along with IVC thrombosis progress,^[30]^ its treatments were still limited. CDT/PMT are still wildly used practice patterns,^[4,19]^ however for bleeding complications and contraindications,^[31]^ they aren’t suitable for all patients. The present study found out that it may be a better option to remove IVCFs with thrombus under protection of suprarenal IVCFs when clot burden can’t be reduced to the acceptable volume.

It is one of the suprarenal IVCFs placement indications based on guidelines written by the Society of Interventional Radiology that thrombus extending above previous placed infrarenal filter.^[1]^ For the suprarenal IVC’s larger diameter and shorter length,^[32]^ multiple perceived risks may be associated with suprarenal IVCFs placement, including migration,^[33]^ fracture, more challenging retrieval. Renal vein thrombosis and resultant renal dysfunction.^[34,35]^ While some recent studies showed a promising result of suprarenal IVCFs^[36,37]^ which may be consistent with our result that suprarenal IVCFs prevented thrombus detachment and major of them were removed safely. Nonetheless more attentions should be paid before their placement, most studies including present study reported extra technique were needed during retrieval.^[36,37]^ In present study, even several measures were applied, still 1 patients had to keep suprarenal IVCF permanent.

The mainstay of Treatments for IVC thrombus including IVCFs thrombus is still anticoagulation.^[30]^ The thrombus burden could be reduced after an additional period of anticoagulation, which may facilitate later retrieval.^[19,36]^ Both former studies did not mention which drugs were used for addition anticoagulation,^[19,36]^ while present study chose factor Xa inhibitions (Rivaroxaban) for additional anticoagulation, which was reported more effective in anticoagulation.^[38]^ Most cases thrombus burden reduced after a period of anticoagulation in present study confirmed former studies, but 2 infrarenal IVCFs thrombus burden couldn’t decrease even after a long period of anticoagulation (54 days and 101 days). It was worth noting that both cases presented IVC occlusion, which made our center consider venous hemodynamics maybe the key part, but it still need more data to be confirmed. What is more, the additional period of anticoagulation for thrombus burden reduction was longer than 30 days^[19,36]^ (the present study also had this situation), which means this management could not fit all kinds filter thrombus.

Several important limitations must be noted in the present study. For the nature of retrospective study, data collection and DSA image might not be complete or free of bias. Furthermore, some factors like types of IVCFs, the time of filters retrieval, the period of additional anticoagulation, failed to be standardized. Finally, although there was no major complication, long term follow-up were missing in present study.

## 5. Conclusion

The large-size IVCFs thrombus may be break off during retrieval, and infrarenal IVCFs with large-size thrombus could be remove safely with suprarenal IVCFs protection when thrombus regression failed or contraindications existed. When the cava were not occlusion, the thrombus trapped in filters could be reduced with an additional period of anticoagulation.

## Author contributions

**Conceptualization:** Xiangdong Liu.

**Data curation:** Yaodong Dou.

**Funding acquisition:** Yanrong Zhang.

**Investigation:** Xintong Luo.

**Resources:** Liang Li.

**Writing – original draft:** Pengkai Cao.

**Writing – review & editing:** Yunsong Li.
